# Non-Adherence to Medication in Outpatient Setting in Nigeria: The Effect of Employment Status

**DOI:** 10.5539/gjhs.v6n3p37

**Published:** 2014-02-14

**Authors:** Senanu Okuboyejo

**Affiliations:** 1 Department of Computer and Information Science, Covenant University, Ota, Nigeria

**Keywords:** outpatient, non-adherence, interventions, healthcare, Nigeria

## Abstract

**Background:**

Non-adherence to prescribed medication and health regimen has been identified as responsible for poor health outcomes. This study investigates the reasons for medication non-adherence in outpatient setting among malaria patients in Nigeria.

**Methods:**

This research adopted quantitative research methods. A well-structured questionnaire was completed by 440 respondents with minimum age of 18 years. The aim of the questionnaire was to get respondents’ reasons for non-adherence to medication. The demographic details of the respondents were also captured.

**Results:**

Age, gender, educational level, marital status and medication payment were found not to influence non-adherence while employment was a significant variable. Respondents also indicated fear of death, nauseating smell of drugs, religious beliefs, the side effects of medication, the fear of taking counterfeit drugs or drugs that are past their expiry dates as also responsible for non-adherence.

**Conclusion:**

The results highlighted reasons for poor adherence in southwest Nigeria. Interventions can be targeted towards these reasons.

## 1. Introduction

Health challenges present arguably the most significant barrier to sustainable global development. Disease and the lack of adequate preventative care take a significant toll on both developing populations and economies. According to the United Nations Report (2008) and the World Health Statistics (2010), the health related Millennium Development Goals (MDGs) include: reducing child mortality, improving maternal health and combating Human Immunodeficiency Virus/Acquired Immune Deficiency Syndrome (HIV/AIDS), Malaria, Tuberculosis and other diseases. In 2008, there were an estimated 243 million cases of malaria causing 863,000 deaths; mostly of children under five (5) years old (World Malaria Report, 2009). Access to treatment (especially artemisinin-based combination therapy) was inadequate in all countries surveyed in 2007 and 2008. By the end of 2012, the UN reported broad and significant progress in the achievement of the MDGs across countries and regions (United Nations, 2012). There was an increase in access to treatment for people living with HIV across the different regions; tuberculosis incidence rates were reduced while global malaria deaths have also declined. The estimated incidence of malaria decreased globally by seventeen per cent (17%) since year 2000. Over the same period, malaria-specific mortality rates have decreased by twenty-five per cent (25%). Reported malaria cases fell by more than fifty per cent (50%) between 2000 and 2010 in 43 of the 99 countries with ongoing malaria transmission. Despite the great progress recorded with the MDGs across regions, the targets have not been reached.

The most common contributors to the disease burden in Nigeria are Malaria, Tuberculosis (TB) and HIV/AIDS (Labiran et al., 2008). These diseases are chronic, infectious/non-infectious, and highly prevalent. The current goals and objectives of the health sector include: reduction of the disease burden from HIV/AIDS and control and eradication of malaria and tuberculosis. Adherence to long-term therapy in outpatient setting is required to reduce prevalence of these diseases (WHO, 2003). Adherence is generally described as the extent to which patients take medications as prescribed by their health care providers (Osterberg & Blaschke, 2005). Rates of adherence for individual patients are usually reported as the percentage of the prescribed doses of the medication actually taken by the patient over a specified period. Some investigators have further refined the definition of adherence to include data on dose taking (taking the prescribed number of pills each day) and the timing of doses (taking pills within a prescribed period) (Osterberg & Blaschke, 2005).

Non-adherence to prescribed medication and health regimen has been identified as responsible for poor health outcomes. Most researchers agree that at least 50% of patients fail to receive the full benefit of prescribed drugs due to inadequate adherence (Rogers & Bullman, 1995). Non-adherence and poor adherence to long-term therapies severely compromise the effectiveness of treatment, making this a critical issue in population health both from the perspective of quality of life and of health economics. There are studies that have attempted to identify the predictors of adherence and non-adherence. These variables are behavioral and dynamic; and have been classified as provider behaviors, health system factors and patient attributes. In (Brown, 1994; Wright, 1998; Scopp, 2000), provider behaviors were discussed and identified. These research works identified that factors affecting interaction and communication between healthcare providers and patients were the key determinants of adherence. Health system variables include availability and accessibility of services, support for education of patients, data collection and information management, provision of feedback to patients and healthcare providers, and training provided to health service providers (WHO, 2003).

Age, sex, income, marital status, ethnic background, and race are patient characteristics but have not been found to be direct determinants of adherence (Haynes et al., 1980; Kaplan & Simon, 1990). However, a strong relationship exists between medication adherence and perceptions of disease factors and beliefs about treatment. According to Freitas et al. (2010), the predictors of adherence are classified into three categories: (a) demographic, social and economic factors; (b) factors related to the disease and the therapeutic regimen prescribed; and (c) factors related to the patient’s relationship with health professionals and services.

Adherence to malaria treatment, that is taking all the doses that are given, is very important for successful malaria treatment outcome. Non-adherence to malaria treatment can lead to re-oocurence of the ailment, the parasites becoming resistant to the drug, so in future the drug will be less effective against the parasites. Critical to patients’ adherence is good communication between healthcare providers and their patients (http://labspace.open.ac.uk). Adherence to malaria medication in patients has been linked to knowledge of malaria, access to information on medication for malaria, perceived benefit from the medication, and perceived barriers to treatment. In Brazil, the low compliance with malaria treatment probably explains the large number of Plasmodium vivax malaria relapses observed in the past years. Pereira et al. (2011) studied the proportion of patients adhering to the Plasmodium vivax malaria treatment with chloroquine and primaquine in the dosages recommended by the Brazilian Ministry of Health. Patients who were being treated for Plasmodium vivax malaria with chloroquine plus primaquine were eligible for the study. On the seventh day of taking primaquine, they were visited at their home and were interviewed. The patients were classified as probably adherent, if they reported having taken all the medication as prescribed, in the correct period of time and dosage, and had no medication tablets remaining; probably non-adherent, if they reported not having taken the medication, in the correct period of time and dosage, and did not show any remaining tablets; and certainly non-adherent, if they showed any remaining medication tablets. The study showed that 242 of the 280 patients reported having correctly followed the prescribed instructions and represented a treatment adherence frequency (CI95%) of 86.4% (81.7%-90.1%).

Diala et al. (2013) conducted a cross-sectional study in peri-urban and rural communities of Nasarrawa and Cross-River states in Nigeria to understand the perceptions of intermittent preventive treatment of malaria in pregnancy (IPTp) and barriers to adherence. Study instruments were based on the socio-ecological model and its multiple levels of influences, taking into account individual, community, societal, and environmental contexts of behaviour and social change. The study found that systems-based challenges (stockouts, lack of provider knowledge of IPTp protocols) coupled with individual women’s beliefs and lack of understanding of IPT contribute to low uptake and adherence. Many pregnant women are reluctant to seek care for an illness they do not have. Those with malaria often prefer to self-medicate through drug shops or herbs, though those who seek clinic-based treatment trust their provider and willingly accept medicine prescribed.

The aim of this paper is to identify and evaluate some of the reasons for medication non-adherence among malaria patients in outpatient setting in major cities of southwest Nigeria.

## 2. Method

This research is the first phase of a cross-sectional survey on medication adherence. The study was carried out in some major cities in southwest Nigeria. We investigated socio-demographic characteristics and reasons that have been identified from literature (Nemes et al., 2004; Vik et al., 2004; Jackson et al., 2005; Vlasnik et al., 2005; Erah & Arute, 2008; Olowookere et al., 2008; Adisa et al., 2009; Uzochukwu et al., 2009; Ekwunife et al., 2010; and Farley et al., 2010) and listed in Tables 1 and 2 for possible correlation with medication adherence. This research adopted quantitative research methods. The survey instrument combined the instruments used in previous research studies on medication adherence and found in (Erah & Arute, 2008; Uzochukwu et al., 2009; Ekwunife et al., 2010). Provision was made for respondents to indicate other reasons not captured by the identified reasons. The questionnaire also captured socio-demographic details such as sex, age range, and level of education, employment status, occupation and health plan of respondents. Copies of the instrument were administered to a convenient and random sample.

**Table 1:** Socio-demographic characteristics of respondents

Variable	Frequency	Percent
**Age**		
18-27	144	32.7
28-37	156	35.5
38-47	93	21.1
48-57	17	3.9
58-67	4	0.9
Missing Value	26	5.9
**Total**	**440**	**100.0**
**Marital Status**		
Single	194	44.1
Married	220	50.0
Divorced	1	0.2
Widowed	2	0.5
Missing Value	23	5.2
**Total**	**440**	**100.0**
**Gender**		
Male	201	45.7
Female	205	46.6
Missing Value	34	7.7
**Total**	**440**	**100.0**
**Employment Status**		
Employed	293	66.6
Self-employed	37	8.4
Unemployed	80	18.2
Missing value	30	6.8
**Total**	**440**	**100.0**
**State/Location**		
Lagos	88	20.0
Ogun	117	26.6
Ondo	79	18.0
Osun	12	2.7
Oyo	111	25.2
Ekiti	3	0.7
Others	15	3.4
Missing value	15	3.4
**Total**	**440**	**100.0**
**Educational Level**		
Primary school and below	4	0.9
Secondary school	64	14.5
Graduate	227	51.6
Postgraduate	127	28.9
Missing value	18	4.1
**Total**	**440**	**100.0**
**Medical Bill Payment**		
Self	261	59.3
NHIS	56	12.7
Family	82	18.6
Welfare/Charity	2	0.5
Employer	31	7.0
Missing value	8	1.8
**Total**	**440**	**100.0**

**Table 2:** Factors influencing medication adherence rate

Label	Question	N (Yes)	N (No)	%(Yes)	%(No)
**MAB1**	I do not understand the need for medication	40	400	9.1	90.9
**MAB2**	I do not believe the usefulness of medication	42	398	9.5	90.5
**MAB3**	I do not get support and motivation	71	369	16.1	83.9
**MAB4**	I do not want to make medications-taking a habit	281	159	63.9	36.1
**MAB5**	Medication is not readily available	78	362	17.7	82.3
**MAB6**	I do not have a good relationship with the doctor	56	384	12.7	87.3
**MAB7**	I do not keep my medications in sight and within reach.	117	323	26.6	73.4
**MAB8**	I am not satisfied with the treatment	81	359	18.4	81.6
**MAB9**	I lack confidence in the doctor	48	392	10.9	89.1
**MAB10**	My medication is expensive	76	364	17.3	82.7
**MAB11**	I do not like the side effects of the medication	212	228	48.2	51.8
**MAB12**	I do not want to mix medication with alcohol/any other substance	172	268	39.1	60.9
**MAB13**	I feel worse when I take my medications	54	386	12.3	87.7
**MAB14**	The medication/treatment has no effect	53	387	12.0	88.0
**MAB15**	I feel better before I finish the medication/treatment	292	148	66.4	33.6
**MAB16**	The duration of the treatment is too long	119	321	27.0	73.0
**MAB17**	I have to take too many medicines/drugs	88	352	20.0	80.0
**MAB18**	I am lazy at taking medicines	159	281	36.1	63.9
**MAB19**	I find it difficult to take my medicines according to prescription e.g during meals, every 8 hours, with a lot of liquid, etc	156	284	35.5	64.5
**MAB20**	I have no time, always busy	102	338	23.2	76.8
**MAB21**	I have strong religious or cultural beliefs regarding health and medication	90	350	20.5	79.5
**MAB22**	I do not like taking medications	167	273	38.0	62.0
**MAB23**	I forget	146	294	33.2	66.8

The administration of the survey followed an approach which was meant to ensure the validity of the responses to questions on medication adherence behavior. All study participants were informed of their rights by means of an informed consent letter that briefly stated the purpose of the study, its significance, issues of confidentiality, and rights of the respondent. Each participant was also informed before the interview took place that she/he was under no obligation to participate. The informed consent was read aloud to the hearing of prospective participants. Furthermore, participants were informed of their right of confidentiality and that at no time should they disclose any information with which they felt uncomfortable. To maintain confidentiality, there was no identifying information on any questionnaire. The data collected were entered into Statistical Package for Social Sciences (SPSS) version 16.0 software for analysis. The Chi-square test, linear regression and independent *t*-test were used for evaluating factors associated with medication adherence.

### 2.1 Research Design

A cross sectional survey was used for this study. The study was designed to gather information about the study population at a particular point in time. Data were collected by adopting quantitative methods using a paper-based questionnaire and conducting semi-structured interviews. Before distributing the questionnaire and conducting the interviews, all study participants were informed of their rights, benefits and risks by means of an informed consent letter that briefly stated the purpose of the study, its significance, issues of confidentiality, and rights of the respondent. Each participant was also informed before the interview took place that she/he was under no obligation to participate. The informed consent was read aloud to the hearing of prospective participants. Furthermore, participants were informed of their right of confidentiality and that at no time should they disclose any information with which they felt uncomfortable. To maintain confidentiality, there was no identifying information on any questionnaire. The survey items were also translated to the native language “Yoruba dialect” for the benefit of those who could not read in English. The respondents returned their completed questionnaire to the researcher and research assistants immediately. The study population was adult Nigerians who were on any form of malaria medication in outpatient settings. Our intention is to understand the adherence behaviour of individuals on medications in outpatient setting and design an intervention based on the findings, hence, the study included individuals who were on any form of malaria medication. The inclusion criteria for participants in the study were 1) 18 years of age or older; 2) must be required to take some form of medication for the treatment of malaria; 3) the medication must be over a period of time e.g two days; 4) the medication must be taken in an outpatient setting, the individual must not be on admission in the hospital. The study sample was a combination of random and convenience sample selected from some major cities – Abeokuta, Ado, Akure, Ibadan, Lagos and Oshogbo - in the southwest region of Nigeria based on the above criteria. The sample size was calculated based on the following assumption: since we were using a random sample, our intention was to maximize responses from as many subjects as possible. Hence, confidence level was set at 95%, precision level (margin of error) is also given as +/- 5%; we projected a 90% response rate, 0.9, and calculated our sample to be 427 respondents. This sample size is to estimate the proportion of adherent to non-adherent respondents.

## 3. Results

### 3.1 Statistics and Data Analysis

Six hundred (600) questionnaires were distributed; four hundred and sixty (460) returned questionnaires were received. Ten (10) questionnaires were dropped as a result of incomplete responses for each of the measurement items. Ten (10) questionnaires were dropped because the respondents gave invalid response of “not taking any form of medication”. This left four hundred and forty (440) questionnaires for the statistical analysis, which represented a 73% valid return rate, this is statistically significant. This was calculated via a ratio of the returned and valid questionnaires and the administered questionnaires.

The sample is made up of two hundred and one (201) males and two hundred and five (205) females while thirty-four respondents did not indicate their gender. One hundred and fifty six (156) respondents are between 28-37 years (35.5%), accounting for a major part of the entire population, one hundred and forty four (144) respondents are between 18-27 years (32.7%), ninety three (93) are between 38-47 years (21.1%), seventeen (17) fall into 48-57 age range (3.9%), 0.9% (4) respondents are in the 58-67 age range while twenty six (26) respondents did not disclose their age range (5.9%). One hundred and ninety four (194) respondents are single; two hundred and twenty (220) are married, one (1) divorced and two (2) widowed while twenty three (23) respondents did not disclose their marital status. Also, a greater portion of the respondents pay for their medication themselves. The socio-demographic characteristics of the respondents are represented in Table 1.

Table 2 presents the reasons investigated and estimated as responsible for poor adherence to medication in respondents who have been or currently on prescribed malaria treatment.

From the responses, 281 (63.9%) respondents do not follow their treatment plan because they do not want to make it a habit (MAB4), 117 (26.6%) respondents do not keep their medication in sight and within reach (MAB7), 212 (48.2%) respondents do not like the side effects of medications (MAB11), 172 (39.1%) respondents do not want to mix medication with alcohol or any other substance (MAB12), 292 (66.4%) respondents indicate they feel better before they finish the medication (they experience some level of wellness before completing the required dosage) (MAB15), 119 (27.0%) respondents feel the duration of the medication is too long (MAB16), 159 (36.1%) respondents are lazy at taking medications (medicines) (MAB18), 156 (35.5%) respondents find it difficult to take medicines according to prescription (MAB19), 102 (23.2%) respondents indicate a busy schedule (MAB20), 167 (38.0%) respondents do not like taking medicines (MAB22), while 146 (33.2%) respondents forget to take their drugs (MAB23).

Furthermore, 40 (9.1%) respondents say they do not understand the need for medication (MAB1), 42 (9.5%) respondents do not believe the usefulness of medication (MAB2), 71 (16.1%) respondents would follow their treatment plan if they get support and motivation (MAB3), 78 (17.7%) respondents indicated non-availability of medication (MAB5), 56 (12.7%) respondents do not have a good relationship with their doctors (MAB6), 81 (18.4%) respondents are not satisfied with their treatment, hence do not take their medication (MAB8), 48 (10.9%) respondents lack confidence in their doctor (MAB9), 76 (17.3%) respondents complained of high cost of medication, 54 (12.3%) respondents explained that they feel worse when they take medications (MAB13), 53 (12.0%) respondents believe the medication has no effect, 88 (20.0%) respondents have to take too many drugs/medicines (MAB17), while 90 (20.5%) respondents have strong religious or cultural beliefs regarding health and medication (MAB21).

In the bi-variate analysis carried out, the reason with the highest response rate (MAB15) represented by “I feel better before I finish the medication” (experiencing some level of wellness before completing the required dosage) was used to measure medication adherence. Respondents’ age, marital status, educational level, employment status, gender and medication payment were all positively correlated to medication adherence. A chi-square test was performed and no relationship was found between age and medication adherence [X^2^ (4, N=440) = 0.858, p= .931]; marital status and medication adherence [X^2^ (1, N=440) = 0.533, p= .465]; educational level and medication adherence [X^2^ (1, N=440) = 1.529, p= .216]; gender and medication adherence [X^2^ (1, N=440) = 0.202, p= .653]. Meanwhile, a positive significant relationship was found between respondents’ employment status and medication adherence [X^2^ (1, N=440) = 4.875, p= .027]. The results are presented in Table 3.

**Table 3:** Chi-Square Statistics for “I feel better before I finish the medication”

Demography Attribute	X^2^ Value	Df (*Degree of freedom*)	Significance *(2-sided)*
**Age**	0.858	4	0.931
**Marital Status**	0.533	1	0.465
**Educational Level**	0.529	1	0.216
**Employment status**	4.875	1	0.027
**Sex**	0.202	1	0.653
**Medication Payment**	1.602	2	0.449

## 4. Discussion

The study investigated the reasons for non-adherence to malaria medication in outpatient setting. Age, marital status, educational level, gender, medication payment did not cause any variation in medication adherence behavior while employment status caused a significant variation i.e being employed makes me non-adherent.

The factor “I feel better before I finish the medication” had the highest affirmative response and thus established as a prominent factor. This implies many of the respondents stop their anti-malaria medication before completing the prescribed dosage because they experience feelings of wellness after taking some of the medications. This factor has also been established from literature. Other reasons given include the fear of death, nauseating smell and taste of drugs, religious beliefs, the side effects of medication (dizziness, nausea, itching), quantity of drugs to take per time, the fear of taking fake (counterfeit) drugs or drugs that are past their expiry dates. Some respondents also prefer liquid drugs (syrup) to tablets, complained about the distance of health institutions, drug stores (where original drugs are sold) and healthcare personnel to their homes. Others do not believe nor have confidence in the competence of the healthcare practitioners due to the contrary opinions of such personnel while some feel getting the required support and motivation from people who care and matter to them will make them adhere to their medications.

### 4.1 Implications for Clinical Practice

The findings from this work can be used in several ways in clinical practice. Employment status was found to have a negative influence on medication adherence rate. This may not make patients initiate or complete prescribed treatment regimen. Being employed (including being self-employed and the nature of job) connotes a busy lifestyle where medication may not fit in. For instance, individuals may find it difficult to take time off work to complete medical treatment such as injections and rehab therapy, fear of stigmatization or public knowledge of one’s health may prevent an individual from taking his medication at work, the side effects of some medication (e.g drowsiness) may also influence medication non-adherence. Health care providers should take into consideration the employment status and nature of job of patients before recommending, prescribing or initiating a treatment regimen. Prescriptions should fit into the patient’s work style.

### 4.2 Implications for Employers

Employers should create policies that encourage employees to take matters regarding their health status important. There should not be penalty for missing work based on health grounds. Employers should also take part or full responsibility for the financial implications of medical treatment. This will go a long way in motivating employees to visit health care providers and initiate prescribed treatment regimen.

This study was conducted in southwest Nigeria; medication adherence interventions such as reminding, monitoring, informing and educating interventions could be designed to target these factors for outpatients living in this region. Instances include the use of electronic caps for drugs such that they record each time the patient opens the drug box, the use of mobile phones to send alerts to patients to remind them of their medication, mobile phones can also be used to inform and educate patients of the causes and preventive measures of ailments and diseases. Health care providers, personnel and institutions need to be aware of these factors. Efforts should be made to educate and inform patients on the need to complete their medication irrespective of how they feel. Incentives and rewards can be introduced into treatments. Patients who successfully complete their medication can be rewarded in the most suitable way.

## 5. Conclusion

This study is a cross-sectional study of factors influencing medication non-adherence among malaria outpatients in southwest Nigeria. These can give the requirements for the design of tailored and targeted interventions to control and influence adherence to medication. Such interventions could be evaluated to measure their effectiveness and efficiency. Adherence to medication will help to curb and reduce the prevalence of malaria, improve population health while reducing health risk factors. This will move Nigeria closer towards achieving the millennium development goal (MDG) on healthcare for all.
